# A clinical pharmacology study of the novel, selective urate reabsorption inhibitor dotinurad in outpatients

**DOI:** 10.1007/s10157-020-01857-0

**Published:** 2020-02-18

**Authors:** Tatsuo Hosoya, Kazuki Furuno, Shingo Kanda

**Affiliations:** 1 Jikei University School of Medicine, 3-25-8, Nishi-Shimbashi, Minato-ku, Tokyo, 105-8461 Japan; 2 Clinical Research Department, Mochida Pharmaceutical Co., Ltd., 1-22 Yotsuya, Shinjuku-ku, Tokyo, 160-0004 Japan

**Keywords:** Hyperuricemia, Gout, Selective urate reabsorption inhibitor (SURI), URAT1 inhibitor, Dotinurad, FYU-981

## Abstract

**Background:**

Dotinurad is a novel, selective urate reabsorption inhibitor (SURI), which reduces serum uric acid levels by selective inhibition of the urate transporter 1 (URAT1). The Japanese guideline for the management of hyperuricemia and gout recommends that drug selection should be based on classification of hyperuricemia as a fundamental principle. However, there may be some cases where this principle is not observed. We investigated the pharmacodynamics and safety of dotinurad in outpatients with uric acid overproduction or uric acid underexcretion type.

**Methods:**

This was a multicenter, open-label, forced titration study. Patients were classified as uric acid overproduction or underexcretion type. Study treatment was initiated at 0.5 mg/day, followed by dose titration to the estimated maximum dose of 4 mg/day over 14 weeks. The primary endpoint was urinary uric acid excretion at each 24-h urine collection.

**Results:**

A total of 26 hyperuricemic patients with or without gout were enrolled in the study and assigned to the uric acid overproduction group (overproduction group) or the uric acid underexcretion group (underexcretion group). Although urinary uric acid excretion, the primary endpoint, tended to be slightly greater in the overproduction group, no notable difference was noted between the two hyperuricemic types. Neither type had noteworthy safety concerns associated with dotinurad.

**Conclusion:**

The results of the study demonstrated no relevant differences between the hyperuricemic types in terms of pharmacodynamic action and safety of dotinurad.

**Electronic supplementary material:**

The online version of this article (10.1007/s10157-020-01857-0) contains supplementary material, which is available to authorized users.

## Introduction

Hyperuricemia is considered to be a pathological condition causing uric acid deposition diseases (e.g., gouty arthritis, tophus), and is defined by serum uric acid levels exceeding 7.0 mg/dL [1, 2]. Urinary calculi constitute another uric acid deposition disease. It is common knowledge that the pathological condition of hyperuricosuria facilitates calculus formation [3] and a formed calculus lodged in the urinary tract often causes severe pain. Calculi are known to be associated with elevated serum creatinine levels and decreased estimated glomerular filtration rate (eGFR), and may affect renal function [4]. Correcting the hyperuricemic condition and maintaining uric acid levels ≤ 6.0 mg/dL (the treatment goal) are critical for the prevention of uric acid deposition diseases [1].

This study was planned and conducted in accordance with the Japanese guideline for the management of hyperuricemia and gout, second edition [2], which was the latest edition when designing and conducting the trials. The guideline was revised in 2018 [1]; however, this paper describes based on the second edition. The guideline classified hyperuricemia into overproduction type, underexcretion type, and combined type, each, respectively, accounting for hyperuricemia in approximately 12%, 60%, and 25% of patients [2]. Moreover, the guideline recommended drug selection based on the classification of hyperuricemia. Drug therapies for hyperuricemia are broadly divided into two classes: xanthine oxidoreductase inhibitors (XOIs), which reduce uric acid production, and uricosuric drugs, which increase urinary uric acid excretion [2]. In principle, it is proposed that uricosuric drugs should be used in underexcretion-type patients and XOIs should be used in overproduction-type patients [2]. However, it is reported that improvement in serum uric acid levels and symptoms of gout during treatment may influence the lifestyle of patients including dietary habits, thus leading to a change in the disease type [2]. This is probably because changes in body purine intake during diet therapy as well as changes in the total body uric acid pool resulting from treatment are responsible for changes in urinary uric acid excretion, one of the indices used for classification of hyperuricemia. Furthermore, due to the potential need to select drugs that diverge from the fundamental principle of the guidelines to avoid side effects, and reports that the rate of utilization of hyperuricemia classification by type is 35.6% in clinical practice [5], dotinurad may be administered to overproduction-type patients. When used in overproduction-type patients, a uricosuric agent is considered to excessively increase urinary uric acid excretion [2]. Moreover, it is reported that increased urinary uric acid excretion is associated with higher frequencies of urinary calculus formation [6]. In line with these considerations, the guideline recommends that uricostatic drugs should be used in overproduction-type patients with increased urinary uric acid excretion [1].

Dotinurad, a novel selective urate reabsorption inhibitor (SURI), reduces serum uric acid levels by selectively inhibiting urate transporter 1 (URAT1), which is expressed on the proximal renal tubules and is responsible for reabsorption of uric acid [7]. In contrast, benzbromarone, the most common uricosuric drug in Japan, has inhibitory effects not only on URAT1 but also on the organic anion transporter (OAT) 1 and OAT3, both of which are responsible for uric acid secretion in urine, and the ATP-binding cassette transporter G2 (ABCG2), which is responsible for uric acid secretion from the intestinal tract [7]. Both agents reduce serum uric acid levels by increasing urinary uric acid excretion; however, dotinurad, an SURI, is expected to be more efficient in reducing serum uric acid levels than benzbromarone, which inhibits multiple transporters.

Completed phase 2 studies of dotinurad demonstrated a dose-dependent serum uric acid lowering effect and a favorable safety profile [NCT#02344862, NCT#02416167]. In consideration of the possibility that dotinurad, a drug that reduces uric acid levels by stimulating uric acid excretion, would be used in overproduction-type patients, we conducted a clinical pharmacology study to compare the pharmacodynamics and safety of dotinurad between overproduction-type and underexcretion-type under hospitalized control conditions [NCT#02837198]. In previous pharmacological studies, diet and lifestyle were controlled and these effects on urinary uric acid exclusion were eliminated; the results showed no significant differences between the two types in terms of percent change in serum uric acid level, amount of urinary uric acid excretion, and safety of dotinurad. On the other hand, the present study was conducted in outpatients to reflect the patient’s actual diet and lifestyle because we consider it necessary to evaluate the pharmacodynamics and safety of dotinurad under conditions similar to those in actual clinical practice.

## Methods

### Study design

This was a multicenter, open-label, forced titration, clinical pharmacology study conducted at 10 medical institutions in Japan.

### Inclusion and exclusion criteria

The inclusion criteria included the following: male patients aged 20 years and older at the time of informed consent; a serum uric acid level on the first day of the run-in period ≥ 7.0 mg/dL (patients with a history of gout or with gouty tophi), ≥ 8.0 mg/dL (patients with any of hypertension, diabetes mellitus, and metabolic syndrome under treatment or follow-up), or ≥ 9.0 mg/dL (patients without any of the above conditions under treatment or follow-up) in reference to the Japanese guideline [2]; patients who were classified as the overproduction or underexcretion type on the first day of the run-in period; outpatients; and patients who appropriately completed a 24-h urine collection on the final day of the run-in period. Incidentally, females were not included in this study because female had a menstrual cycle, it was considered difficult to implement appropriate urine collection, and many hyperuricemia patients were males [1].

The exclusion criteria included the following: patients with unresolved gouty arthritis in the 14 days before the day of assignment/registration; patients who used any serum uric acid-lowering agent during the specified period between the first day of the run-in period and the day of assignment/registration; patients who changed the dosing regimen of any drug that may affect serum uric acid levels between 14 days before the first day of the run-in period and the day of assignment/registration; patients with a history of allergy to potassium citrate/sodium citrate hydrate combination preparations; patients with a history of urinary calculus or in whom ultrasound or plain radiography of the abdomen on the start day of the run-in period revealed a urinary calculus; patients who previously received dotinurad; patients with HbA1c ≥ 8.4%; patients with serum alanine aminotransferase (AST) and/or aspartate aminotransferase (ALT) ≥ 100 IU/L, eGFR < 30 mL/min/1.73 m^2^, systolic blood pressure ≥ 180 mmHg or diastolic blood pressure ≥ 110 mmHg on the first day of the run-in period; and patients whose participation in the study was considered unsuitable in the opinion of the investigator.

Patients who were on uric acid-lowering medication underwent at least 28 days of washout after informed consent. Patients who were on medication that could affect serum uric acid levels or safety underwent at least 14 days of washout after informed consent.

### Treatment

**Fig. 1:**
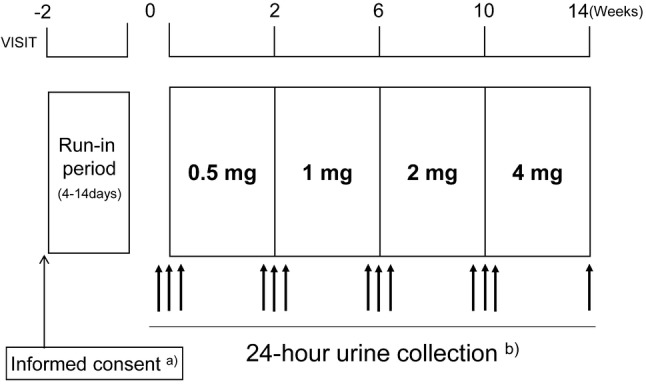
Study scheme. **a** Patients underwent at least 28 days (on uric acid lowering medication) or 14 days (on medication that could affect efficacy evaluation or safety) of washout after informed concent, then started the run-in period. **b** During the study, thirteen 24-h urine collections were conducted for pharmacodynamic evaluation: 2 days before treatment at each dose, on the day and the day after starting treatment at each dose, and on the day before the end of treatment

Figure 1 shows the study scheme. Prior to initiation of any study-related procedures, written informed consent was obtained from all patients. Patients were assigned to either the overproduction group or the underexcretion group at a 1:1 ratio based on the classification of hyperuricemia.

The study adopted dose titration to minimize the risk of gouty attacks due to a rapid fall in serum uric acid levels [1]. Dotinurad was administered at a starting dose of 0.5 mg/day for two weeks and then at 1, 2, and 4 mg/day for four weeks each (a total of 14 weeks of treatment).

During the study, thirteen 24-h urine collections were conducted for pharmacodynamic evaluation (Fig. 1): two days before treatment at each dose, on the day and the day after starting treatment at each dose, and on the day before the end of treatment. A portable urine sampling container URINEMATE®-P (Sumitomo Bakelite Co., Ltd.) was provided to each patient, so that they could easily collect urine samples on their own, without mistakes.

To minimize the risk of urinary calculus formation associated with increased urinary uric acid excretion, patients concomitantly received urine alkalization therapy with potassium citrate/sodium citrate hydrate combination preparations during the treatment period, unless the investigator decided that it was unnecessary.

### Hyperuricemia classification

On the first day of the run-in period, a blood sample and a 60-min urine sample were collected and urinary extraction of uric acid [*E*_UA_ (mg/kg/h)] and uric acid clearance [*C*_UA_ (mL/min)] were determined to identify the patient’s disease type according to the following criteria: (i) overproduction type, *E*_UA_ > 0.51 and *C*_UA_ ≥ 7.3; (ii) underexcretion type, *E*_UA_ < 0.48 or *C*_UA_ < 7.3; (iii) combined type, *E*_UA_ > 0.51 and *C*_UA_ < 7.3; and (iv) normal type, 0.48 ≤ *E*_UA_ ≤ 0.51 and *C*_UA_ ≥ 7.3. Patients classified as the combined or normal type were excluded from the study. The hyperuricemic type was determined in accordance with the then current Japanese guideline for the management of hyperuricemia and gout [2], when the study was planned.

### Pharmacodynamic evaluations

The primary endpoint was urinary uric acid excretion at 13 time points when 24-h urine samples were collected. The secondary endpoints were urinary uric acid/urinary creatinine levels at the same 13 time points and serum uric acid levels at selected time points (Weeks 2, 6, 10, and 14 and at the end of treatment).

### Safety evaluations

The investigator evaluated adverse events (AEs) and safety based on vital signs, 12-lead electrocardiography, laboratory tests, and physical examination. AEs were coded by the System Organ Class and Preferred Term (MedDRA version 21.0) and the causal relationship to the study drug, and the severity and seriousness of each event were evaluated. An adverse drug reaction (ADR) was defined as an AE that was considered to be related to the study drug.

### Statistical analyses

The target number of patients was determined to be 12 patients per group, in consideration of the feasibility of the study and a necessary sample size for pharmacodynamic assessment.

Pharmacodynamic analyses were performed on the full analysis set (FAS) consisting of patients who received at least one dose of the study drug and had at least one pharmacodynamic parameter evaluated after treatment.

The summary statistics of urinary uric acid excretion as the primary endpoint, and the summary statistics of secondary endpoints, namely, urinary uric acid/urinary creatinine levels and serum uric acid levels were also calculated for each group and their changes were plotted over time.

Safety analyses were performed on the safety population (SP) consisting of patients who received at least one dose of the study drug and had evaluable safety information after treatment. The number and proportion of patients with AEs and the number of AEs were calculated and tabulated. SAS software, version 9.3 (SAS Institute, Cary, NC, USA), was used in statistical analyses.

## Results

### Patient flowcharts and baseline characteristics

**Fig. 2:**
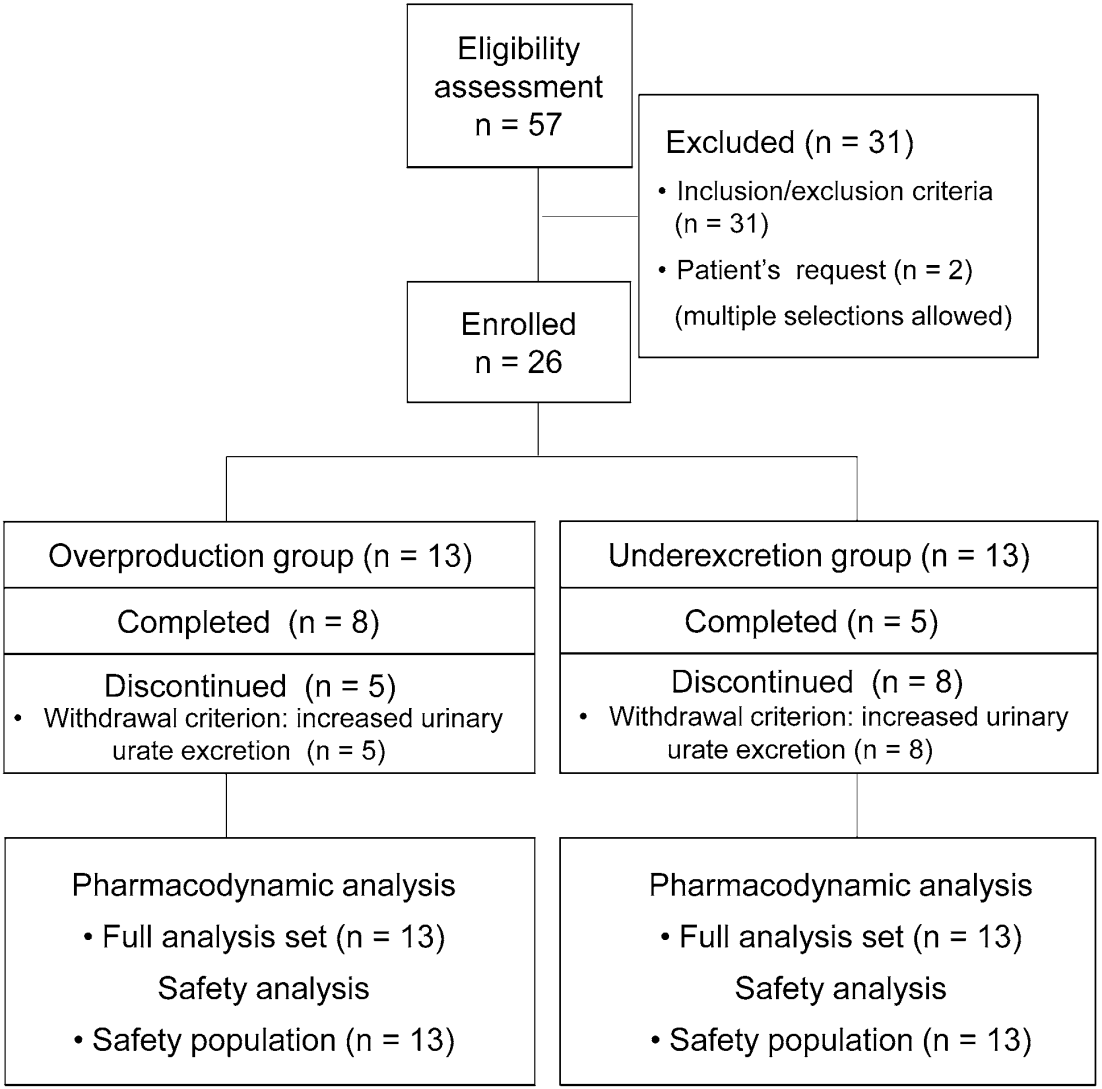
Flowcharts of patients in this study

In this study, 57 patients were screened and 31 were excluded. Common reasons for exclusion were failure to meet the inclusion criteria and falling under exclusion criteria. The remaining 26 patients were assigned to either the overproduction group (*n* = 13) or the underexcretion group (*n* = 13). Eight patients in the overproduction group and five in the underexcretion group completed the study, and the remaining patients discontinued the study for the same reason: they all met a withdrawal criterion that urinary uric acid excretion on the day after starting treatment at each dose is ≥ 15% greater than that on the day of starting treatment at each dose (Fig. 2).

The FAS and SP comprised 13 patients each in the overproduction and underexcretion groups. (Fig. 2).

The demographic and other baseline characteristics in the FAS were as follows: mean ages of 50.5 and 61.6 years; mean urinary uric acid excretion of 648.84 and 609.58 mg; mean eGFR of 79.5 and 66.5 mL/min/1.73 m^2^; and the percentage of patients with a history of gouty arthritis of 100.0 and 61.5%; mean Urinary uric acid/Creatinine ratio of 0.457 and 0.411, in the overproduction and underexcretion groups, respectively. No relevant differences in the other characteristics were noted between the groups. The mean serum uric acid level before treatment was 8.31 mg/dL in the overproduction group and 8.29 mg/dL in the underexcretion group (Table 1).

**Table 1:** Summary of the baseline characteristics

Characteristics			Overproduction group (*n* = 13)	Underexcretion group (n = 13)	Total (n = 26)	P value^c^
Sex	No of patients (%)	Male	13 (100.0)	13 (100.0)	26 (100.0)	– –
		Female	0 (0.0)	0 (0.0)	0 (0.0)	
Age (years)	Mean ± SD		50.5 ± 10.3	61.6 ± 10.0	56.0 ± 11.4	0.012^d^
Height (cm)	Mean ± SD		169.00 ± 6.10	169.40 ± 6.96	169.20 ± 6.41	1.000^d^
Body weight (kg)	Mean ± SD		72.62 ± 14.63	76.14 ± 12.34	74.38 ± 13.38	0.317^d^
BMI (kg/m^2^)	Mean ± SD		25.43 ± 5.06	26.44 ± 3.32	25.94 ± 4.23	0.249^d^
Urinary uric acid excretion (mg)	Mean ± SD		648.84 ± 111.84	609.58 ± 244.36	629.21 ± 187.26	0.118^d^
Serum uric acid level (mg/dL)	Mean ± SD		8.31 ± 0.56	8.29 ± 0.60	8.30 ± 0.57	0.877^d^
eGFR (mL/min/1.73m^2^)^a^	Mean ± SD		79.5 ± 11.1	66.5 ± 10.7	73.0 ± 12.6	0.009^d^
Urinary uric acid/Creatinine ratio	Mean ± SD		0.457 ± 0.076	0.411 ± 0.067	0.434 ± 0.074	0.118^d^
Treatment history	No of patients (%)	No	3 ( 23.1)	3 ( 23.1)	6 ( 23.1)	1.000^e^
		Yes	10 ( 76.9)	10 ( 76.9)	20 ( 76.9)	
History of gouty arthritis	No of patients (%)	No	0 ( 0.0)	5 ( 38.5)	5 ( 19.2)	0.039^e^
		Yes	13 (100.0)	8 ( 61.5)	21 ( 80.8)	
Gouty Tophi	No of patients (%)	No	13 (100.0)	13 (100.0)	26 (100.0)	–
		Yes	0 ( 0.0)	0 ( 0.0)	0 ( 0.0)	
Drinking habit^b^	No of patients (%)	No	4 ( 30.8)	3 ( 23.1)	7 ( 26.9)	1.000^e^
		Yes	9 ( 69.2)	10 ( 76.9)	19 ( 73.1)	

^a^eGFR (mL/min/1.73 m^2^) = 194×Serum creatinine^−1.094^ × Age^−0.287^

^b^Definition of drinking habit: consumption of alcohol on more than 3 days of the week and consumption of more than 500 mL of beer or 60 mL of whisky in a day

^c^Two-sided significance level of 15%,

^d^Wilcoxon rank-sum test

^e^Fisher’s exact test

### Pharmacodynamics

#### Primary endpoint

**Fig. 3:**
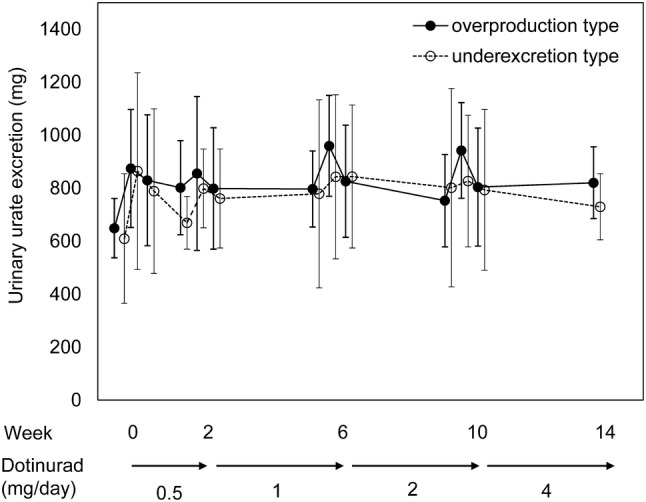
Time course of urinary uric acid excretion. Error bars indicate the standard deviation (SD)

The urinary uric acid excretion (mean ± standard deviation [SD]) before treatment with 0.5 mg, on the day treatment started, on the day after starting treatment, and before dose titration to 1 mg was 648.84 ± 111.84, 874.12 ± 222.98, 829.48 ± 246.84, and 801.65 ± 177.50 mg, respectively, in the overproduction group and 609.58 ± 244.36, 864.21 ± 371.25, 788.88 ± 310.64, and 669.13 ± 99.44 mg, respectively, in the underexcretion group. There was no meaningful difference between the groups in terms of changes in urinary uric acid excretion from before treatment to the end of treatment with 0.5 mg. During dose titration, a transient increase followed by a decrease in urinary uric acid excretion, as seen at the start of treatment, was observed in both groups. A similar pattern of changes was observed when the dose was titrated to 1, 2, and 4 mg. In both groups, urinary uric acid excretion transiently increased and then declined to approximately 800 mg (Table 2, Fig. 3). The difference of urinary uric acid excretion between before treatment and end of treatment was + 171.49 mg in the overproduction group, and + 120.10 mg in the underexcretion group.

**Table 2:** Urinary uric acid excretion at each time point

	Time point	Urinary uric acid excretion (mg)			
		No. of patients	Mean ± SD	Two-sided 95% CI for the mean	
				Lower limit	Upper limit
*Overproduction group*					
0.5 mg	Before treatment	13	648.84 ± 111.84	581.25	716.42
	Day treatment started	13	874.12 ± 222.98	739.37	1008.86
	Day after starting treatment	13	829.48 ± 246.84	680.32	978.64
1 mg	Before dose increase	11	801.65 ± 177.50	682.41	920.90
	Day of dose increase	11	855.10 ± 290.36	660.03	1050.17
	Day after dose increase	11	798.82 ± 229.25	644.81	952.83
2 mg	Before dose increase	8	796.61 ± 143.45	676.69	916.54
	Day of dose increase	8	959.53 ± 190.56	800.21	1118.84
	Day after dose increase	8	825.84 ± 211.81	648.76	1002.91
4 mg	Before dose increase	8	752.63 ± 174.31	606.90	898.35
	Day of dose increase	8	941.95 ± 180.68	790.90	1093.00
	Day after dose increase	8	803.94 ± 222.57	617.86	990.01
	End of treatment	8	820.33 ± 135.34	707.18	933.47
*Underexcretion group*					
0.5 mg	Before treatment	13	609.58 ± 244.36	461.92	757.25
	Day treatment started	13	864.21 ± 371.25	639.86	1088.55
	Day after starting treatment	13	788.88 ± 310.64	601.16	976.59
1 mg	Before dose increase	9	669.13 ± 99.44	592.70	745.57
	Day of dose increase	9	798.77 ± 149.10	684.16	913.38
	Day after dose increase	9	761.02 ± 186.76	617.47	904.58
2 mg	Before dose increase	8	778.68 ± 354.69	482.15	1075.20
	Day of dose increase	8	843.11 ± 309.42	584.43	1101.79
	Day after dose increase	8	843.79 ± 269.59	618.40	1069.17
4 mg	Before dose increase	7	801.60 ± 374.43	455.31	1147.89
	Day of dose increase	7	826.63 ± 248.06	597.21	1056.04
	Day after dose increase	7	793.29 ± 303.07	512.99	1073.58
	End of treatment	5	729.68 ± 124.60	574.97	884.39

#### Secondary endpoints

**Fig. 4:**
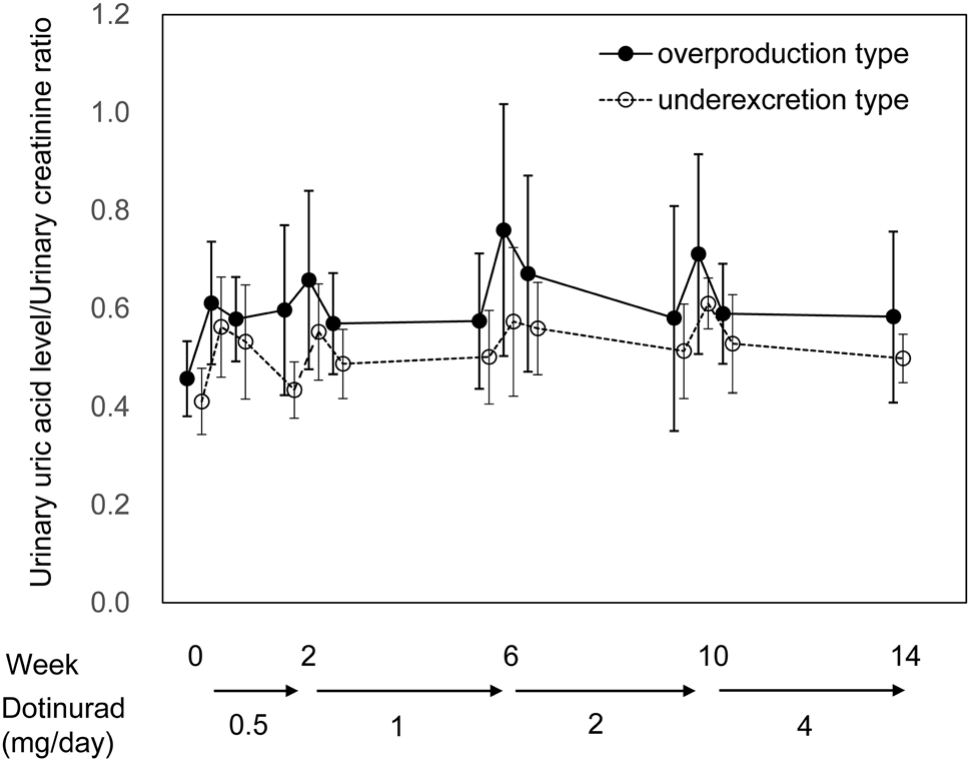
Time course of Urinary uric acid/Creatinine ratio. Error bars indicate the standard deviation (SD)

The urinary uric acid/urinary creatinine ratio (mean ± SD) before treatment with 0.5 mg, on the day treatment started, on the day after starting treatment, and before dose titration to 1 mg was serum 0.457 ± 0.076, 0.611 ± 0.125, 0.578 ± 0.086, and 0.597 ± 0.173 mg/dL, respectively, in the overproduction group and 0.411 ± 0.067, 0.562 ± 0.102, 0.532 ± 0.116, and 0.434 ± 0.057 mg/dL, respectively, in the underexcretion group. During dose titration, a transient increase followed by a decrease in urinary uric acid/creatinine ratio, as seen at the start of treatment, was observed in both groups (Table 3, Fig. 4). This tendency was similar to that for urinary uric acid excretion (primary endpoint). The urinary uric acid/creatinine ratio at end of treatment was 1.28 times in the overproduction group and 1.21 times in the underexcretion group compared to those at the start of treatment.

**Table 3:** Urinary uric acid/creatinine ratio at each time point

	Time point	Urinary uric acid/creatinine ratio			
		No. of patients	Mean ± SD	Two-sided 95% CI for the mean	
				Lower limit	Upper limit
*Overproduction group*					
0.5 mg	Before treatment	13	0.457 ± 0.076	0.411	0.503
	Day treatment started	13	0.611 ± 0.125	0.536	0.687
	Day after starting treatment	13	0.578 ± 0.086	0.526	0.630
1 mg	Before dose increase	11	0.597 ± 0.173	0.481	0.713
	Day of dose increase	11	0.658 ± 0.182	0.535	0.780
	Day after dose increase	11	0.569 ± 0.103	0.500	0.639
2 mg	Before dose increase	8	0.574 ± 0.138	0.458	0.689
	Day of dose increase	8	0.760 ± 0.257	0.545	0.975
	Day after dose increase	8	0.671 ± 0.200	0.504	0.838
4 mg	Before dose increase	8	0.580 ± 0.229	0.388	0.771
	Day of dose increase	8	0.711 ± 0.204	0.541	0.881
	Day after dose increase	8	0.589 ± 0.102	0.503	0.674
	End of treatment	8	0.583 ± 0.174	0.437	0.728
*Underexcretion group*					
0.5 mg	Before treatment	13	0.411 ± 0.067	0.370	0.451
	Day treatment started	13	0.562 ± 0.102	0.501	0.624
	Day after starting treatment	13	0.532 ± 0.116	0.462	0.602
1 mg	Before dose increase	9	0.434 ± 0.057	0.390	0.477
	Day of dose increase	9	0.552 ± 0.098	0.477	0.628
	Day after dose increase	9	0.487 ± 0.070	0.433	0.540
2 mg	Before dose increase	8	0.501 ± 0.095	0.421	0.580
	Day of dose increase	8	0.573 ± 0.151	0.447	0.699
	Day after dose increase	8	0.559 ± 0.094	0.480	0.638
4 mg	Before dose increase	7	0.513 ± 0.096	0.424	0.603
	Day of dose increase	7	0.610 ± 0.052	0.562	0.658
	Day after dose increase	7	0.528 ± 0.100	0.436	0.620
	End of treatment	5	0.498 ± 0.049	0.437	0.559

**Fig. 5:**
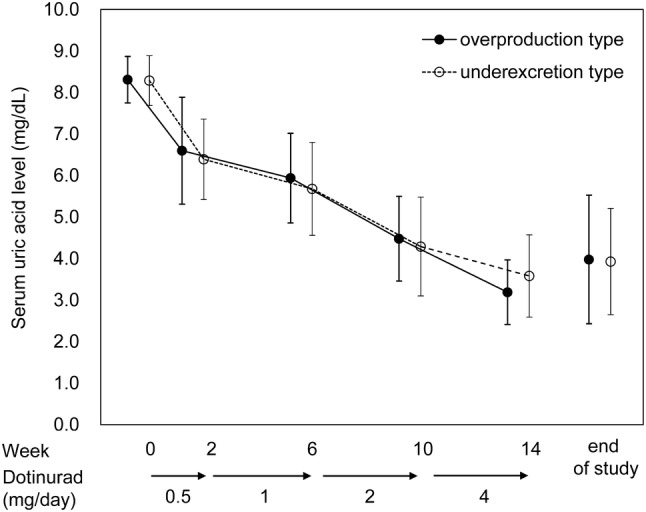
Time course of the serum uric acid level. Error bars indicate the standard deviation (SD)

No relevant difference in changes in serum uric acid levels was noted between the groups at any time points. The serum uric acid level (mean ± SD) in the overproduction and underexcretion groups was 3.19 ± 0.78 and 3.58 ± 0.99 mg/dL, respectively, at Week 14 and 3.98 ± 1.55 and 3.93 ± 1.28 mg/dL, respectively, at the end of treatment (Fig. 5).

#### Safety

The incidence of AEs in the overproduction and underexcretion groups was 15.4 and 38.5%, respectively. The incidence of ADRs in the overproduction and underexcretion groups was 7.7 and 15.4%, respectively. The ADRs included ‘low density lipoprotein increased’ in the overproduction group, and ‘musculoskeletal discomfort’ and ‘oedema peripheral’ in the underexcretion group (each with one event in a single patient). In either group, no AEs occurred in more than one subject, and all reported AEs were mild in severity. Serious adverse events, AEs leading to treatment discontinuation (Table 4), gouty arthritis, or urinary calculus were not reported in either group.

**Table 4:** Summary of adverse events

	Overproduction type			Underexcretion type		
	(*n* = 13)			(*n* = 13)		
	No. of patients	Incidence (%)	No. of events	No. of patients	Incidence (%)	No. of events
AEs	2	( 15.4)	4	5	( 38.5)	5
ADRs	1	( 7.7)	1	2	( 15.4)	2
SAEs excluding deaths	0	( 0.0)	0	0	( 0.0)	0
AEs leading to treatment discontinuation	0	( 0.0)	0	0	( 0.0)	0

## Discussion

Dotinurad, an SURI, reduces serum uric acid levels by increasing urinary uric acid excretion. Researchers report a positive correlation between urinary uric acid excretion and the prevalence of urinary calculi in patients with gout [6]. The use of uricosuric agents in hyperuricemic patients of the overproduction type is thought to cause a marked increase in urinary uric acid excretion [2], though it has yet to be proven in randomized controlled studies. However, uricosuric drugs are assumed to be prescribed to patients with hyperuricemia of the overproduction type in actual clinical practice. One of the reasons is a reported change in the disease type during treatment. In our previous inpatient study, some patients experienced a change in their disease type from the overproduction type to the underexcretion type during screening period of the study [NCT#02837198]. In light of these findings, evaluation of the pharmacodynamics and safety of dotinurad in each hyperuricemia type has great significance, under the assumption that the dotinurad may be used irrespective of the hyperuricemia type.

This study evaluated the pharmacodynamics and safety of dotinurad administered for 14 weeks in hyperuricemia outpatients with or without gout of the overproduction and underexcretion types.

Some points should be considered before interpreting the results of the study. Of 26 patients enrolled, 13 (5 in the overproduction group and 8 in the underexcretion group) discontinued the study for the same reason: they all met a withdrawal criterion that urinary uric acid excretion on the day after starting treatment at each dose (dose increase) is ≥ 15% greater than that on the day of starting treatment at each dose (dose increase). This criterion was used in this study to minimize the risk of urinary calculus formation in patients in whom an increase in urinary uric acid excretion was noted on the day after starting treatment, on the basis of the findings in the inpatient study that urinary uric acid excretion increased on the day on which treatment started, and decreased on the following day. A urinary calculus may be caused not only by increased urinary uric acid excretion but also by decreased urine output/insufficient water intake and persistently acid urine [1]. These factors elevate urinary uric acid levels or lower the solubility of urinary uric acid, thereby triggering the deposition of uric acid crystals in the urinary tract. In this study, urine output in both groups was not meaningfully different (Supplement 1).

**Fig. 6:**
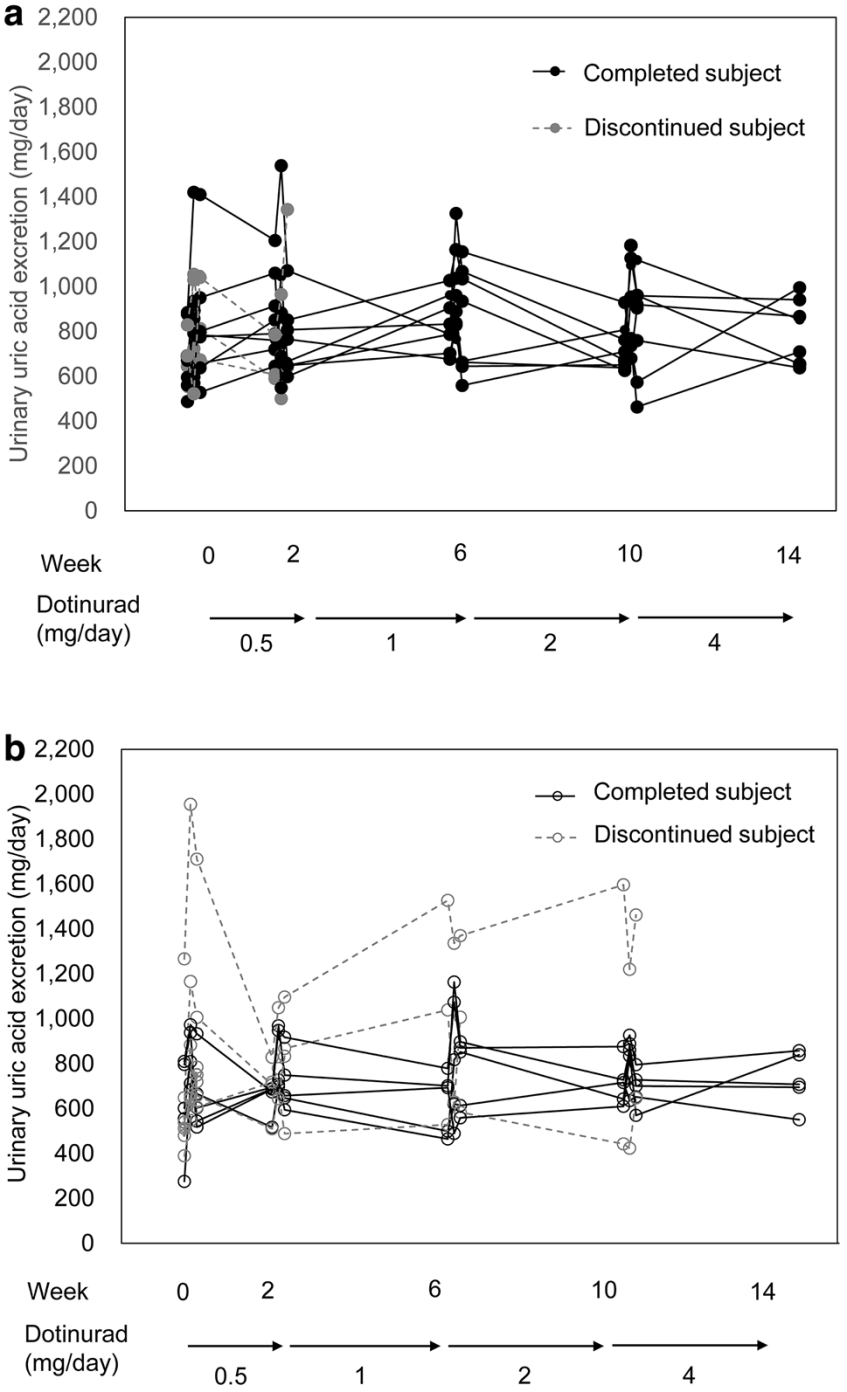
Individual data on urinary uric acid excretion. **a** Overproduction type. **b** Underexcretion type

Individual review of changes in urinary uric acid excretion over time revealed that urinary uric acid excretion in patients who discontinued, with some exceptions, was not biased toward higher levels compared to patients who completed the study (Fig. 6). Urinary uric acid excretion at discontinuation substantially exceeded 1000 mg in one patient each in the two groups. To evaluate the risk of urinary calculus formation from another point of view, urinary uric acid levels at discontinuation were reviewed in these two patients. They were 40.1 and 46.4 mg/dL, which did not meaningfully differ from those patients who completed the study. Urine output at discontinuation in both patients (3350 and 3150 mL) substantially exceeded 1500 mL, the upper limit of daily urine output in normal individuals [8]. Based on these findings, polyuria could explain the increased urinary uric acid excretion observed in both patients.

The two patients had preexisting polyuria, which was likely to be inherent. The other 11 patients included those who experienced an increase in urinary uric acid excretion for three days in a row (before treatment, the day treatment started, after starting treatment) but their urinary uric acid excretion levels were not high; and those who experienced a decline in urinary uric acid excretion on the day of starting treatment followed by an increase on the following day, thus meeting the withdrawal criterion. Withdrawals due to this criterion occurred in both hyperuricemic types.

The results of the study showed no relevant differences in changes in urinary uric acid excretion (primary endpoint) between the overproduction and underexcretion types. Urinary uric acid excretion increased on the day of starting treatment (dose increase) and then decreased on the following day, as observed in the inpatient study [NCT#02837198]. Unlike the inpatient study, the present study in outpatients tended to show a greater variation between patients. This may be explained by greater differences in dietary habit and lifestyle among outpatients.

No relevant differences were observed in the secondary endpoints between the overproduction and underexcretion types. Dotinurad reduced serum uric acid levels similarly in both groups.

During the study, the dose was titrated to 4 mg, the assumed maximum dose of dotinurad. No noteworthy safety concerns arose in each group.

There are some limitations of this study. Firstly, this study was an exploratory study conducted in a small number of patients. Secondary, there are no data available regarding extended treatment with dotinurad in the patients withdrawn from the study. Hence, it is practically impossible to draw a definite conclusion solely from the results of the study regarding changes in urinary uric acid excretion as well as the risk of urinary calculus formation for each hyperuricemic type; however, the results in the study do not indicate, from the perspective of urinary uric acid excretion, that dotinurad therapy clearly increases the risk of urinary calculus formation in overproduction-type patients. Any increase in urinary uric acid excretion after dosing with dotinurad was transient, as observed in the previous inpatient study. Combining the results of the two studies, changes in urinary uric acid excretion after dotinurad administration do not differ in a clinically meaningful way between the overproduction and underexcretion types, and no urinary calculus was found in any patient in either group in this study.

Finally, the 2018 revised guidelines (Third Edition) changed the hyperuricemia classification and defined overproduction type as renal overload type. The renal overload type is further categorized as either overproduction or extra-renal underexcretion type, but the criteria for which are not defined; so, the breakdown of the overproduced group in this study is unknown. The consequences of overproduction type need to be further investigated.

## Electronic supplementary material

Below is the link to the electronic supplementary material.

Supplementary file1 (DOCX 17 kb)
