# Supplementary material for: A clinical pharmacology study of the novel, selective urate reabsorption inhibitor dotinurad in outpatients
Source: Clin Exp Nephrol. 2020 Feb 18;24(Suppl 1):103–11. doi: 10.1007/s10157-020-01857-0 (PMC7066296; doi:10.1007/s10157-020-01857-0)
Supplement: Supplementary file 1 — Supplementary file1 (DOCX 17 kb) [file 10157_2020_1857_MOESM1_ESM.docx]

**Supplement 1** Urine output (mL/day)

|  | Time point | | Urine output (mL/day) | | | | | |
| --- | --- | --- | --- | --- | --- | --- | --- | --- |
|  |  |  | No of patients | Mean | ± | SD | Two-sided 95% CI for the mean | |
|  |  |  |  |  |  |  | Lower limit | Upper limit |
| Overproduction group | 0.5 mg | Before treatment | 13 | 1492.3 | ± | 626.4 | 1113.7 | 1870.9 |
|  |  | Day treatment started | 13 | 1588.5 | ± | 500.9 | 1285.8 | 1891.2 |
|  |  | Day after starting treatment | 13 | 1534.6 | ± | 767.9 | 1070.5 | 1998.7 |
|  | 1 mg | Before dose increase | 11 | 1727.3 | ± | 519.8 | 1378.1 | 2076.5 |
|  |  | Day of dose increase | 11 | 1672.7 | ± | 660.1 | 1229.3 | 2116.2 |
|  |  | Day after dose increase | 11 | 1640.9 | ± | 646.8 | 1206.4 | 2075.5 |
|  | 2 mg | Before dose increase | 8 | 1600.0 | ± | 425.1 | 1244.6 | 1955.4 |
|  |  | Day of dose increase | 8 | 1643.8 | ± | 520.6 | 1208.5 | 2079.0 |
|  |  | Day after dose increase | 8 | 1778.1 | ± | 381.6 | 1459.1 | 2097.1 |
|  | 4 mg | Before dose increase | 8 | 1356.3 | ± | 326.7 | 1083.1 | 1629.4 |
|  |  | Day of dose increase | 8 | 1468.8 | ± | 523.0 | 1031.5 | 1906.0 |
|  |  | Day after dose increase | 8 | 1518.8 | ± | 409.6 | 1176.3 | 1861.2 |
|  |  | End of treatment | 8 | 1362.5 | ± | 288.8 | 1121.1 | 1603.9 |
| Underexcretion group | 0.5 mg | Before treatment | 13 | 1813.5 | ± | 854.6 | 1297.1 | 2329.9 |
|  |  | Day treatment started | 13 | 2173.1 | ± | 1390.7 | 1332.7 | 3013.5 |
|  |  | Day after starting treatment | 13 | 1907.7 | ± | 626.4 | 1529.1 | 2286.3 |
|  | 1 mg | Before dose increase | 9 | 1816.7 | ± | 843.0 | 1168.7 | 2464.6 |
|  |  | Day of dose increase | 9 | 1972.2 | ± | 1038.7 | 1173.8 | 2770.6 |
|  |  | Day after dose increase | 9 | 1877.8 | ± | 819.7 | 1247.7 | 2507.9 |
|  | 2 mg | Before dose increase | 8 | 2106.3 | ± | 1030.4 | 1244.8 | 2967.7 |
|  |  | Day of dose increase | 8 | 1937.5 | ± | 773.6 | 1290.8 | 2584.2 |
|  |  | Day after dose increase | 8 | 1831.3 | ± | 582.4 | 1344.3 | 2318.2 |
|  | 4 mg | Before dose increase | 7 | 2021.4 | ± | 807.7 | 1274.4 | 2768.4 |
|  |  | Day of dose increase | 7 | 1571.4 | ± | 681.2 | 941.4 | 2201.4 |
|  |  | Day after dose increase | 7 | 1707.1 | ± | 726.0 | 1035.7 | 2378.5 |
|  |  | End of treatment | 5 | 1770.0 | ± | 309.4 | 1385.8 | 2154.2 |
